# Nonlinear Optical Properties of Discotic Hexylthiotruxene Derivatives

**DOI:** 10.1021/acsomega.3c06778

**Published:** 2023-11-18

**Authors:** Manish Kumar, Sreekanth Perumbilavil, D. R. Vinayakumara, Alok Goel, Reji Philip, Sandeep Kumar

**Affiliations:** † Department of Mechanical and Materials Engineering, University of Turku, Turku FI-20014, Finland; ‡ Raman Research Institute, C. V. Raman Avenue, Sadashivanagar, Bangalore 560080, India; § Institute of Polymer Nanotechnology (INKA), FHNW University of Applied Sciences and Arts Northwestern Switzerland, School of Engineering, Klosterzelgstrasse 2, Windisch 5210, Switzerland; ∥ Laboratory for Surface Science and Technology, Department of Materials, ETH Zurich, Zürich 8092, Switzerland; ⊥ Department of Chemistry, Nitte Meenakshi Institute of Technology, Yelahanka, Bangalore 560064, India

## Abstract

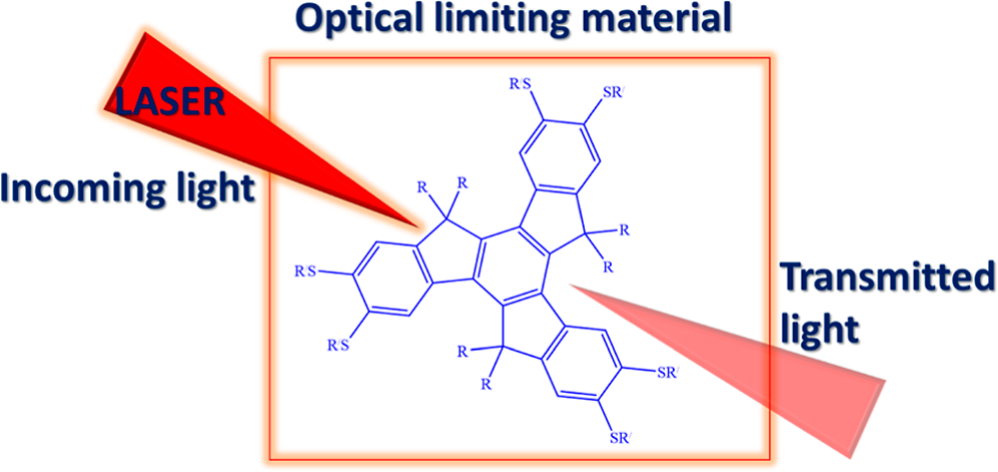

The search for efficient and transparent nonlinear optical (NLO) media has led to the investigation and development of alternative organic optical materials. In this context, a series of new hexylthiotruxene derivatives have been synthesized, and their linear and NLO properties are explored. These truxene derivatives show large NLO absorption due to their C_3_ symmetry, presence of large hyperpolarizability, and extended π-conjugation. Herein, we show that two-photon absorption and three-photon absorption processes are the main cause of nonlinear absorption in these materials under 5 ns and 100 fs excitations at 532 and 800 nm excitations, respectively. The nonlinear absorption coefficients have high values of 2 to 7.9 × 10^–10^ m/W in the nanosecond domain and 2.2 to 7.4 × 10^–21^ m^3^/W^2^ in the femtosecond domain. The corresponding nonlinear absorption cross-section (δ) values and the nonlinear susceptibilities were also calculated from the numerically obtained nonlinear absorption coefficient values. Tailored truxene derivative showed an excellent optical limiting threshold of 4.5 J/cm^2^ and is comparable to or better than most recently reported and benchmark optical limiting materials. Longer alkyl members of the series showed the largest nonlinear absorption in both excitation domains and could be a potential optical limiter.

## 1 Introduction

During the past two decades, nonlinear optics (NLO) have attracted tremendous attention owing to a plethora of possible applications of NLO materials in advanced technologies,^1,2^ image encryption,^3^ optical data storage,^4^ telecommunications,^5^ frequency converters,^6^ and dynamic holography.^7,8^ Compared to the traditional inorganic solids, organic materials have great importance of choice for NLO applications due to their large and fast nonlinearities, high NLO coefficients, high optical damage threshold, ultrafast optical response, flexible molecular design and synthesis, easy processability, and integration into optical devices.^1,9,10^ Organic compounds can be tailored through chemical synthesis, providing a range of NLO properties.^10^ So far, many experimental investigations on NLO properties have been carried out in various liquid and solid mediums and dye-doped systems.^11,12^

Truxene derivatives owing π-conjugated star-shaped systems have received much attention and have been demonstrated to be prospective candidates for various applications, viz., photonic devices,^13^ organic lasers,^14^ organic light-emitting diodes,^15^ solar cells,^16^ single-photon absorption pumped lasers,^17^ and photoelectronic devices.^18^ Extensive research has been conducted on the nonlinearity of star-shaped truxene-based compounds and their use in organic lasers.^19−22^ It is worth noting that truxene is a favorable foundational component for creating two-photon-absorbing chromophores, thanks to its effective two-photon absorption (2PA) capabilities. So far, numerous truxene-based derivatives have been utilized to demonstrate their single-photon absorption (1PA) and 2PA NLO characteristics, mostly on the low lasing threshold and frequency upconverted lasers.^19−23^ Significant work has been devoted to enhancing 2PA nonlinearity via molecular design strategy in truxene derivatives.^19,20,23−25^

To achieve the desired NLO property, a promising strategy is extending the charge-transfer dimension.^26,27^ It has been well documented that extending the π-conjugated organic materials by increasing the number of flexible branches resulted in excellent 2PA properties and 2PA cross-section values.^28,29^ However, extending the branching increases the molecular weight, reducing solubility and transmittance. Therefore, balancing the optimized NLO coefficients and the appropriate compatibility for optical gain media is a significant challenge. However, to date, these truxene derivatives have not been considered for optical limiting purposes in the femtosecond regime. Therefore, it is a necessity for researchers to design and synthesize optical limiters with high transparency at low light intensities and large NLO absorption at higher intensities.

**Scheme 1:**
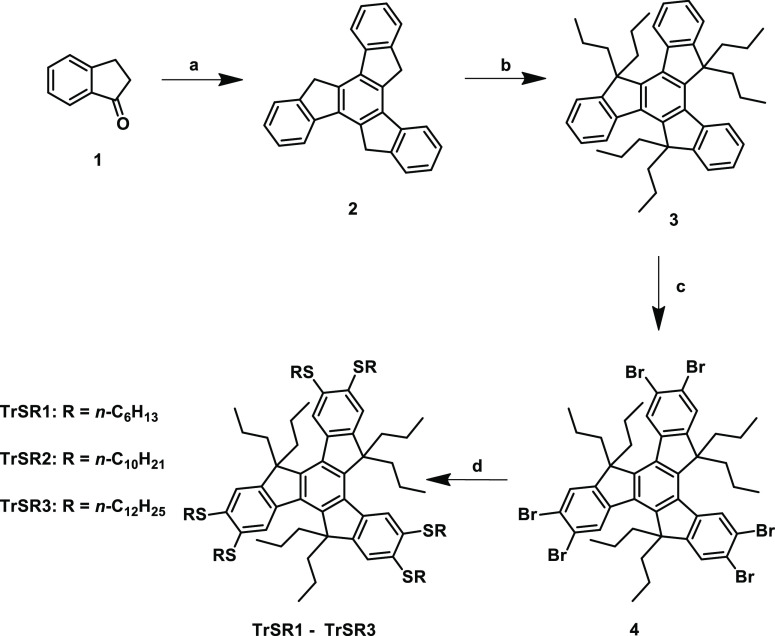
Synthesis of **TrSR1–3**; (a) Conc. HCl, CH_3_COOH, 100 °C 18 h; (b) NaH, C_3_H_7_Br, DMF, RT, 24 h; (c) Br_2_, I_2_, FeCl_3_, CHCl_3_, RT, 6 h; and (d) C_*n*_H_2*n*+1_SH, *t*-BuOK, NMP, 70 °C, 2 h

Truxene is also well-known in the field of liquid crystals. Several discotic liquid crystals (DLCs) have been realized from the truxene core.^30^ DLCs have recently been studied for various optoelectronics applications, such as organic solar cells^31^ and hybrid nanocomposites.^32−35^ We have previously reported the synthesis procedure, detailed characterization, and NLO properties of blue-light emitting truxene liquid crystals at room temperature.^36^ Continuing our previous work, we prepared a new series of truxene derivatives (Scheme 1). These newly synthesized materials do not display liquid crystalline properties, probably due to the steric hindrance of bulky chains in the way region. However, we observed exciting NLO properties in these materials. In this work, we report the design and synthesis of a new series of star-shaped π-conjugated hexathiofunctionalized truxene (HTT) derivatives for NLO applications. We chose and designed these molecules with the intention of (i) the three-dimensional structure of HTT with extended π–π conjugation and (ii) the appropriate molecular weight of HTT to enhance the nonlinearity and linear transmittance. To the best of our knowledge, no studies have been conducted on the NLO properties of truxene-based hexathio derivatives using the nanosecond and femtosecond Z-scan technique.

## 2 Experimental Section

### 2.1 Materials and Methods

2,3-Dihydroinden-1-one, ≥99% (CAS: 83-33-0); 1-bromopropane, 99% (CAS: 106-94-5); 1-hexanethiol, 95% (CAS: 111-31-9); 1-decanethiol, 99% (143-10-2); 1-dodecanethiol, ≥98% (CAS: 112-55-0), potassium *tert*-butoxide, ≥98% (CAS: 865-47-4); and iron(III) chloride, 97% (CAS: 7705-08-0) were purchased from Sigma-Aldrich. NaH, ∼55–60% (CAS: 7646-69-7); bromine, >99% (CAS: 7726-95-6), and iodine, 99% (CAS: 7553-56-2) were purchased from Spectrochem Private Limited. All of the general chemicals and solvents were procured from the local companies and dried using standard protocols before use. To confirm the new compounds, ^1^H NMR spectra in CDCl_3_ were performed using a Bruker AMX 500 MHz spectrometer. The NMR spectra for all molecules are included in the Supporting Information. IR-absorption spectra were recorded by a Shimadzu FTIR-8400 FTIR spectrometer. A Carlo-Erba Flash 1112 analyzer was used for elemental analysis. Geometrical optimization and density functional theory (DFT) calculations were performed using Gaussian 16 Rev. C.01. Theoretical linear and NLO properties were calculated by using the B3PW91/LANL2DZ program, and the open aperture Z-scan technique was used to determine the nonlinear absorption coefficients experimentally. Our Z-scan experimental setup was calibrated using well-known materials such as CS_2_ and C_60_. The CS_2_ is a well-known material with a nonlinear refractive index *n*_2_ = 6 × 10^–14^ cm^2^ W^–1^.^37^ To conduct measurements in the nanosecond domain, a frequency double Nd:YAG laser operating at a wavelength of 532 nm and running at 10 Hz was utilized. On the other hand, a mode-locked Ti–Sapphire laser emitting 800 nm, 100 fs pulses at a repetition rate of 10 Hz was utilized for measurements in the femtosecond range.

### 2.2 Synthesis and Characterization

The synthesis scheme of newly designed truxene derivatives is illustrated in Scheme 1. The HTT derivatives were synthesized from acid-catalyzed trimerization of 1-indanone to truxene (**2**).^38^ Three reactive methylene groups of truxene (**2**) were protected by reacting with 1-bromopropane under a strong basic condition in dry DMF, giving compound (**3**) a high yield.^39^ Then, precursor hexabromotruxene (**4**) was synthesized using execs Br_2_ in the presence of iodine and iron source (FeCl_3_) as a catalyst under dark conditions.^40^ Finally, thiolation of **4** with different alkane-1-thiols under a strong basic condition in dry *N*-methyl-2-pyrroliodne (NMP) afforded the target compounds **TrSR1**–**3**.^41,42^

#### 2.2.1 Synthesis of 10,15-Dihydro-5*H*-diindeno[1,2-*a*:1′,2′-*c*]fluorene (**2**)

2,3-Dihydroinden-1-one, **1** (10 g) was added to a stirred mixture of acetic acid (60 mL) and concentrated hydrochloric acid (30 mL), which was then heated to 100 °C for 18 h. The reaction mass was allowed to warm and poured into ice-cooled water. The yellow precipitate was filtered and washed with water, acetone, and DCM to afford the desired product as a light-yellow solid (yield, 85%). ^1^H NMR (CDCl_3_, 500 MHz, ppm): δ 7.97 (d, *J* = 7.0 Hz, 3H), 7.70 (d, *J* = 7.5 Hz, 3H), 7.51 (t, *J* = 7.5 Hz, 3H), 7.40 (t, *J* = 7.5 Hz, 3H), 4.29 (s, 6H).

#### 2.2.2 Synthesis of 5,5′,10,10′,15,15′-Hexapropyl-10,15-dihydro-5*H*-diindeno[1,2-*a*:1′,2′-*c*]fluorene (**3**)

To a stirred suspension of **2** (1 g, 2.9 mmol) in DMF (50 mL) was added sodium hydride (0.42 g, 17.5 mmol) in two portions at 0 °C. After 30 min, the reaction mixture was warmed to room temperature; 1-bromopropane (3.6 g, 29.2 mmol) was then charged and continued stirring for 24 h. Then, the mixture was separated using DCM and dried under reduced pressure, and the curd was further purified using column chromatography using DCM as an eluent to obtain **3** as a yellow solid (yield, 90%). ^1^H NMR (CDCl_3_, 500 MHz, ppm): δ 8.35 (d, *J* = 7.0 Hz, 3H), 7.92 (d, *J* = 7.0 Hz, 3H) δ 7.48 (d, *J* = 6.0 Hz, 3H), 2.90 (m, 6H), 2.01 (m, 6H), 1.33 (m, 12H), 0.81–0.5 (m, 18H).

#### 2.2.3 Synthesis of 5,5′,10,10′,15,15′-Hexapropyl-2,3,7,8,12,13-hexabromo-10,15-dihydro-5*H*-diindeno[1,2-*a*;1′,2′-*c*]fluorene (**4**)

A mixture of compound **3** (2.0 g, 3.36 mmol), anhydrous FeCl_3_ (10 mg), and a catalytic amount of I_2_ in dry chloroform (20 mL) was stirred for 30 min at room temperature in the dark. The mixture was cooled to 0 °C, and bromine (6.44 g, 40.0 mmol) was added dropwise and stirred for 5 h. Afterward, a dilute sodium thiosulfate solution was added, and the aqueous layer was removed using chloroform. The collected materials were washed with water and brine solution and dried over sodium sulfate. The crude red solid was purified by column chromatography using DCM: MeOH (8:2) as a mobile phase was used to yield a yellow solid (94%). ^1^H NMR (CDCl_3_, 500 MHz, ppm): δ 8.51 (s, 3H), 7.69 (s, 3H), 2.91 (m, 6H), 2.11 (m, 6H), 1.33 (m, 12H), 0.88–0.45 (m, 18H).

#### 2.2.4 Synthesis of (5,5′,10,10′,15,15′-Hexapropyl-10,15-dihydro-5*H*-diindeno[1,2-*a*:1′,2′-*c*]fluorene-2,3,7,8,12,13-hexayl)hexakis(hexylsulfane) (**TrSR1**)

Potassium-*t*-butoxide (0.25 g, 2.24 mmol) was added to the previously stirred solution of hexane-1-thiol (0.27 g, 2.24 mmol) in dry NMP (7 mL) at RT, and the temperature was raised to 100 °C. After 10 min, the mixture was cooled to 70 °C, and hexabromotruxene (0.2 g, 0.18 mmol) was added; it maintained the same condition for another 2 h. After careful TLC examination, distilled water was added to the reaction mixture, separated with diethyl ether, and dried under reduced pressure. The target compound was isolated by using 2% ethyl ether in hexane by column chromatography. ^1^H NMR (CDCl_3_, 500 MHz, ppm): δ 8.44 (s, 3H), 7.30 (s, 3H) δ 3.10 (m, 12H) δ 2.90 (m, 36H), 2.01–1.42 (m, 36H), 1.33–0.53 (m, 36H). Elemental anal. calcd for C_81_H_126_S_6_: C 75.28, H 9.83 S 14.89; experimental C 75.0, H 9.90, S 14.99%.

#### 2.2.5 Synthesis of (5,5′,10,10′,15,15′-Hexapropyl-10,15-dihydro-5*H*-diindeno[1,2-*a*:1′,2′-*c*]fluorene-2,3,7,8,12,13-decyl)hexakis(decylsulfane) (**TrSR2**)

**TrSR2** was synthesized by adopting a similar protocol used for **TrSR1**. ^1^H NMR (CDCl_3_, 500 MHz, ppm): δ 8.45 (s, 3H), 7.33 (s, 3H) δ 3.13 (m, 12H) δ 2.91–2.86 (m, 48H), 2.05–1.32 (m, 90H) 0.98 (m, 18H). Elemental anal. calcd for C_105_H_174_S_6_: C, 77.42; H, 10.77; S, 11.81; experimental C 77.33, H 10.11, S 11.97%.

#### 2.2.6 Synthesis of (5,5′,10,10′,15,15′-Hexapropyl-10,15-dihydro-5*H*-diindeno[1,2-*a*:1′,2′-*c*]fluorene-2,3,7,8,12,13-dodecyl)hexakis(dodecylsulfane) (**TrSR3**)

**TrSR3** was also synthesized by adopting a similar protocol used for **TrSR1–2**. ^1^H NMR (CDCl_3_, 500 MHz, ppm): δ 8.43 (s, 3H), 7.36 (s, 3H) δ 3.12 (m, 12H) δ 2.89–2.66 (m, 48H), 2.50–0.96 (m, 198H). Elemental anal. calcd for C_117_H_198_S_6_: C, 78.19; H, 11.10; S, 10.70; experimental C 78.05, H 11.29, S 10.88%.

## 3 Results and Discussion

### 3.1 Photophysical Properties

**Figure 1:**
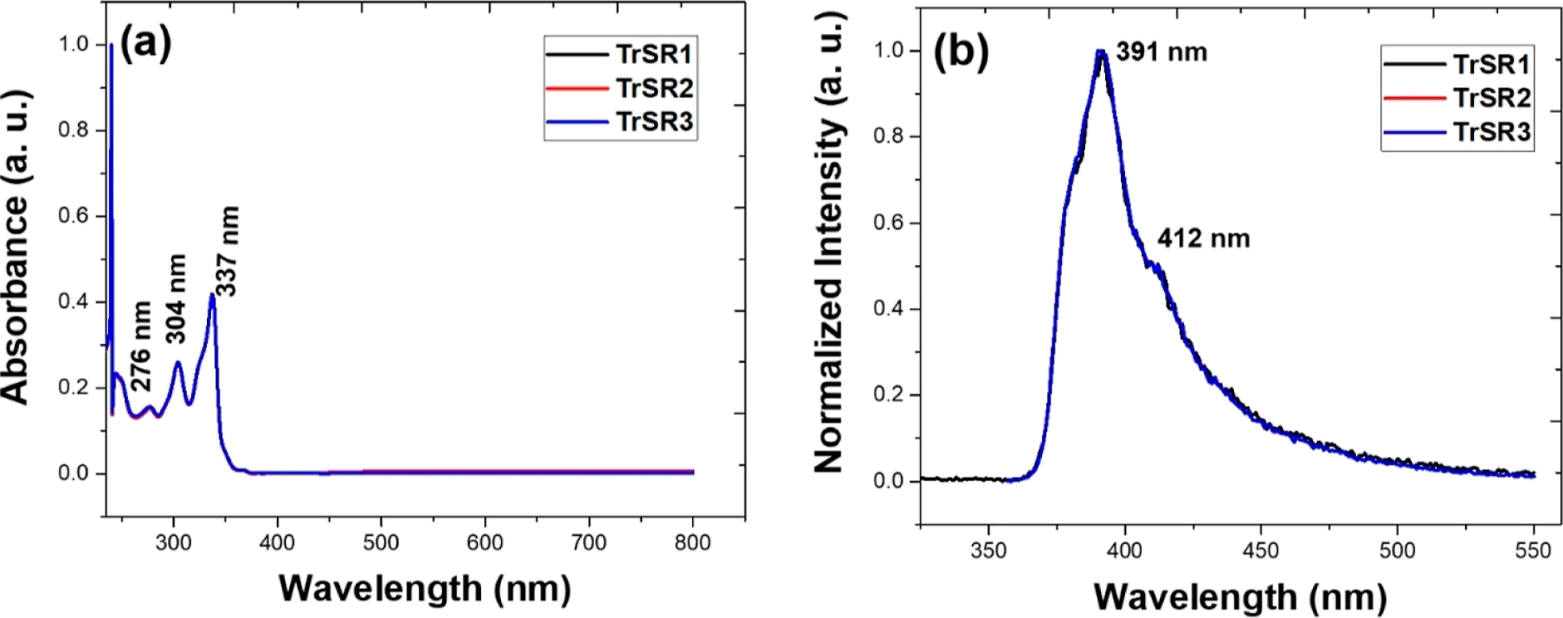
(a) Absorption spectra and (b) photoluminescence spectra of all the truxene derivatives (the concentration of the solution was 0.1 mg in chloroform. For the PL, the excitation wavelength was 330 nm).

The absorption and emission spectra for derivatives **TrSR1**, **TrSR2**, and **TrSR3** are depicted in Figure 1a and b, respectively. Materials with π-conjugation feature a prominent absorption band for π–π* electrons in the UV–visible range. As the effective length of conjugation increases, this band gradually shifts toward longer, more red wavelengths. The absorption spectra of all compounds showed absorbance at 304 nm, corresponding to the truxene chromophore.^43,44^ The high-energy absorption band at 337 nm is assigned to [π →  π*] transitions of hexylthiotruxene (HTT) and vibrionic progression with peaks at 304 and 276 nm.^45^ The small extended spectrum on the low-energy side of the 337 nm peaks is attributed to ground-state charge transfer (CT). On exciting at their absorption maxima, the molecules exhibited emission maxima at around 391 nm (0–0 vibronic transition) and a shoulder at 412 nm (0–1 vibronic transition). As the varied alkyl periphery could not affect their energy of abortion and emission, they resulted in similar linear optical characteristics. However, the PL spectra of all the samples exhibited no excitation wavelength dependence.

### 3.2 Nonlinear Optical Measurements Using the Open Aperture Z-Scan Technique

**Figure 2:**
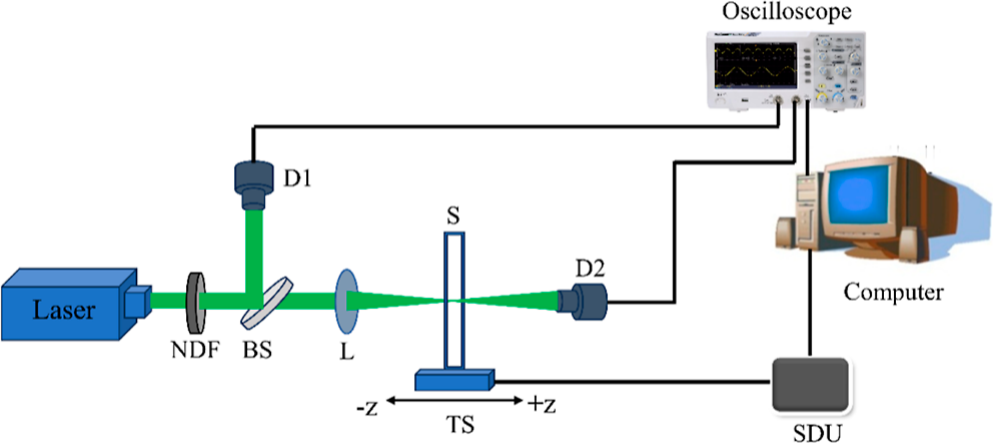
Schematic representation of the automated open aperture Z-scan setup. NDF: neutral density filter, BS: beam splitter, D1, D2: detectors, L: focusing lens, S: sample, TS: motorized translation stage, and SDU: stepper driver unit.

**Figure 3:**
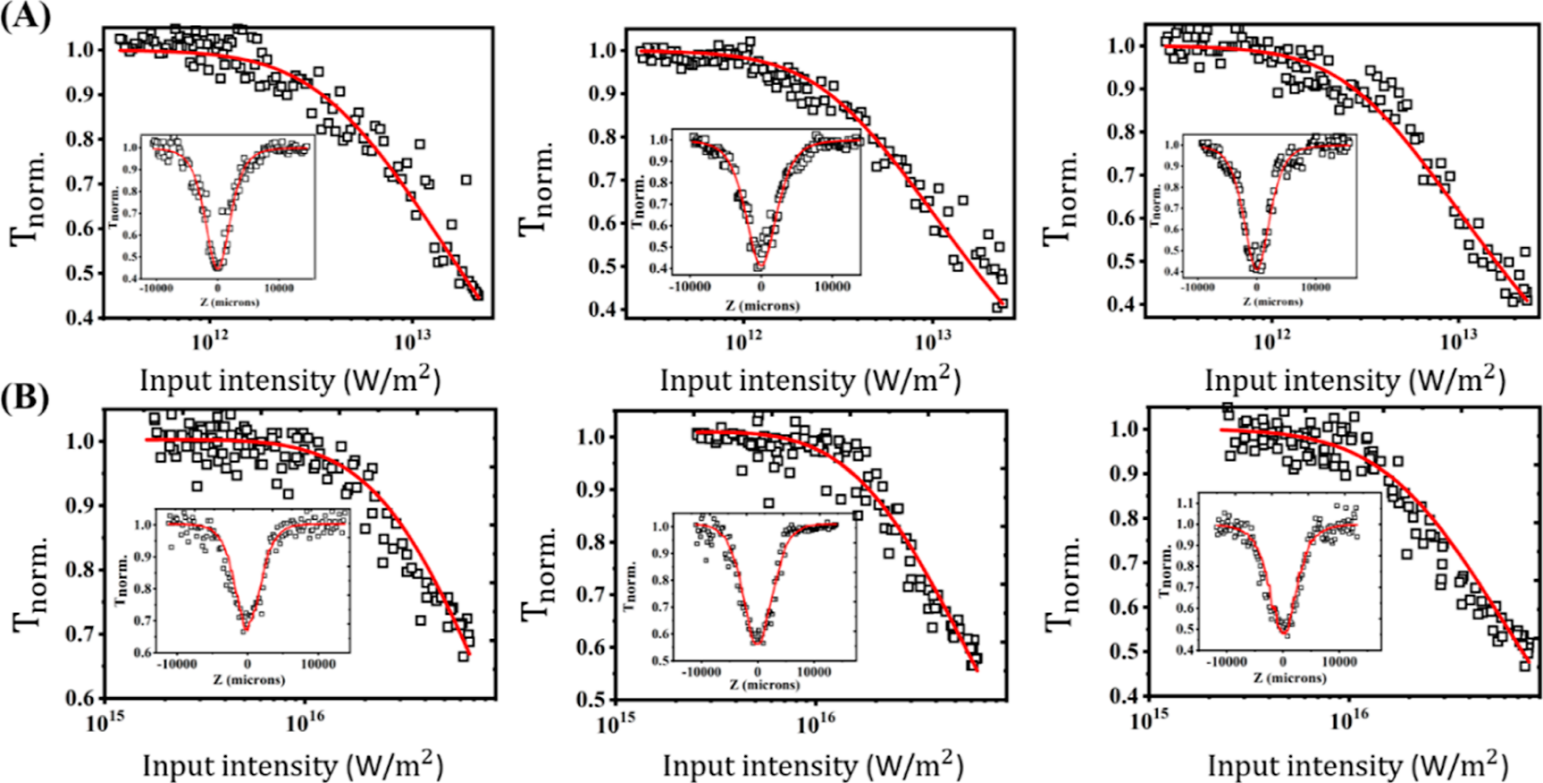
Open aperture Z-scan curves (insets) and corresponding optical limiting curves of (left to right) **TrSR1–3** measured for (A) 523 nm, 5 ns laser pulse excitation, and (B) 800 nm, 100 fs laser pulse excitations, respectively. The laser pulse energy was 50 μJ for nanosecond excitation and 7 μJ for fs excitation. Solid curves are numerical fits, while the symbols are experimental data.

To investigate the NLO absorption and optical power-limiting properties, we employed a conventional open aperture Z-scan experimental technique^10,46^ using 5 ns laser pulses at 532 nm obtained from a frequency-doubled Nd:YAG laser (Minilitte Continuum) and 100 fs laser pulses at 800 nm obtained from a regeneratively amplified Ti–Sapphire laser (TSA-10 Spectra-Physics). A schematic of the setup is shown in Figure 2. All the samples were dispersed in isopropanol in a concentration of about 0.1 mg/mL (about 4.66 × 10^16^, 3.69 × 10^16^, and 3.35 × 10^16^ molecules/cm^3^ for **TrSR1**, **TrSR2**, and **TrSR3**, respectively). The prepared suspensions were stable, and no aggregation or precipitation was observed in all three samples. All the measurements were carried out in a 1 mm thick quartz cuvette, and the measured linear transmissions (LTs) of the samples were ∼89% at 532 nm and ∼92% at 800 nm (including ∼6% reflection at the cuvette). The sample was mounted on a motorized high-precision linear translation stage, which facilitates fine movements in steps of 250 μm. To focus the laser beam, we used a plano-convex lens (*f* = 10.75 cm), which gives a focal spot radius of 18 and 24 μm at 532 and 800 nm, respectively. The sample was then moved along the propagation direction of the laser beam (taken as the *Z*-direction) through the beam focus (*Z* = 0). The transmitted energy at each position was measured by using a pyroelectric laser probe (D2). The intensity at each position can be calculated by using the beam radius at each position. Hence, we get the dependence of transmission on the laser intensity at different positions, which essentially shows the nonlinear absorption characteristics of the material. Using a reference beam and reference detector (D1), we minimized the variation in energy between laser pulses. A detailed description of the experimental setup can be found in refs (47) and (48). To enable the thermal relaxation of the sample, we kept a larger interval between laser pulses (∼1 s). In the nanosecond excitation, this was achieved by externally triggering the Q-switch of the Nd:YAG laser. In the femtosecond excitation case, the laser ran at 10 Hz, and we used a fast shutter to select the laser pulse interval. The input pulse energy used for the experiment was 50 μJ for nanosecond excitation and 7 μJ for femtosecond excitation. The experimentally obtained Z-scan curves and the corresponding optical limiting response measured in **TrSR1**, **TrSR2**, and **TrSR3** for 5 ns and 100 fs excitations are shown in Figure 3. We observed a strong NLO response under both excitation conditions for all three samples. The normalized transmittance strongly depends on the input laser pulse intensity, indicating a typical absorptive nonlinearity. A clear and smooth valley-shaped Z-scan curve indicates reverse saturable absorption. We detected no absorption saturation effect, even at extremely low energy levels, in both excitations.

Given the conditions of our measurements (nanosecond and femtosecond laser excitation and negligible linear absorption at the excitation wavelength) and considering the linear absorption spectra (see Figure 1), 2PA (for nanosecond excitation) and three-photon absorption (3PA) (for femtosecond excitation) will be the nonlinear absorption process leading to the optical limiting process. The pure 2PA and 3PA processes are generally weak, which are the simultaneous absorption of two and three photons mediated via a virtual intermediate state. Since the excited state absorption (ESA) process has also been postulated to play a role in these excitations, and for this reason, the large optical limiting observed in these materials will be due to an effective nonlinear absorption process. Therefore, for this reason, ideally, the nonlinear parameters have been called effective 2PA and 3PA coefficients/cross sections^49^ The best numerical fits to the measured nanosecond Z-scan curves are obtained by considering a 2PA process. For a 2PA process, the nonlinear absorption coefficient can be expressed as
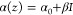
1where α_0_ is the unsaturated linear absorption coefficient. The normalized transmittance, in this case, is given by
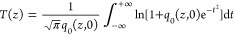
2where *q*_0_ = β(1 – *R*)*I*_0_*L*_eff_, here β is the effective 2PA coefficient, *I*_0_ is the on-axis peak intensity, *L* is the sample length, *R* is the surface reflectivity, and *L*_eff_ is the effective length, which is given by . 2PA cross-section, δ, is related to β through the expression
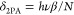
3where *h*ν is the excitation photon energy and *N* is the number of molecules per cm^3^.

The best fit for femtosecond excitation is obtained for a process involving an effective 3PA process. The NLO coefficient in such a case can be written as
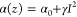
4where α_0_ is the unsaturated linear absorption coefficient, γ is the effective 3PA coefficient, and *I* is the input intensity. The corresponding pulse propagation equation can be expressed as
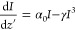
5where *z*′ is the incremental propagation distance. The absorption cross-section corresponding to this 3PA process can be expressed as
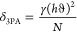
6Numerically calculated NLO coefficients are listed in Table 1. Z-scan results show that NLO absorption strongly depends on the chain length of the **TrSR** molecules in both excitation case, which is observed from the intensity-dependent nonlinear transmission curves, as shown in Figure 3. For instance, in the femtosecond excitation case, at an input pulse intensity of 6 × 10^16^ W/m^2^, **TrSR1** molecules transmit 66% of the LT while **TrSR3** transmits only 46%, which means that the **TrSR3** molecules absorb 20% more light. The strength of the nonlinear absorption coefficients obtained in these materials is in the order **TrSR3** > **TrSR2** > **TrSR1** which directly corelates with the large hyperpolarizability and extended π-conjugation in these materials, which also follows the same order. The measured effective 2PA coefficient (β) values are 2 × 10^–10^ m/W for **TrSR1**, 6.3 × 10^–10^ m/W for **TrSR2**, and 7.9 × 10^–10^ m/W for **TrSR3**. The higher β value corresponds to superior optical limiting properties of the relevant material. By calculating the optical limiting threshold, that is, the input fluence at which the sample’s transmittance drops to 50% of its linear transmittance, we can quantify the performance of these materials as an optical limiter. The optical limiting threshold values calculated for the nanosecond case are 8.1, 6.7, and 4.5 J/cm^2^ for **TrSR1**, **TrSR2**, and **TrSR3**, respectively, revealing that **TrSR3** is a better optical limiter in the lot. This lower optical limiting threshold or the higher nonlinear absorption is due to the larger size and extended π-conjugation and the branching effect of **TrSR3**. In such extended conjugated systems, the electron/energy-transfer process is higher, leading to the enhanced NLO response. Moreover, in the femtosecond regime, the samples demonstrate an optical limiting trend identical to that in the nanosecond regime. **TrSR3** showed a higher 3PA coefficient (γ) of 7.4 × 10^–21^ m^3^/W^2^, which is better than that of donor–acceptor (D–A)-type conjugated polymers,^50^ stilbazolium-like dyes,^51^ carbazole derivatives^52^ and MoS_2_ nanocomposites.^53^ The large 3PA coefficient and cross-section presented in **TrSR3** are due to its longer alkyl chain length and extended π-conjugation (π-electron density along the chain).^54^ Also, the intramolecular charge transfer from the end groups to the conjugated core within the structure of the HTT molecules amplifies their 3PA nonlinear absorption process. The **TrSR3** derivative is appropriate for attenuating high intensity ultrashort and short laser pulses for protecting delicate detectors and the human eye from accidental exposure.

**Table 1:** Nonlinear Optical Absorption Coefficients and Absorption Cross-Section Values Obtained from the Z-Scan Experiments Using 5 and 100 fs Laser Pulses at 532 and 800 nm, Respectively

compounds	excitation conditions							
	5 ns, 532 nm				100 fs, 800 nm			
	laser pulse energy (μJ)	LT (%)	β (m/W)	δ_2PA_ (cm^6^ s^2^)	energy (μJ)	LT (%)	γ (m^3^/W^2^)	δ_3PA_ (cm^6^ s^2^)
**TrSR1**	50	89	2 × 10^–10^	1.603 × 10^–39^	7	92	2.2 × 10^–21^	2.64 × 10^–89^
**TrSR2**	50	91	6.3 × 10^–10^	6.378 × 10^–39^	7	92	4.6 × 10^–21^	7.695 × 10^–89^
**TrSR3**	50	91	7.9 × 10^–10^	8.810 × 10^–39^	7	91	7.4 × 10^–21^	1.363 × 10^–88^

We also estimated the linear and NLO properties using DFT calculations to gain more insights into the NLO properties. We calculated the dipole moment, quadrupole moment, octupole moment, and hexadecapole moment of **TrSR1** in three different solvents: acetone, methanol, and isopropanol. Self-consistent reaction field theory has been extended to include solvent effects using polarizable continuum models. For all calculations based on DFT, the basis set LANL2DZ was used. Optimized geometries have been analyzed for their potential energy surface by performing frequency calculations.

Based on the DFT results and the equations below, we have calculated the values of the dipole moment, quadrupole moment, octupole moment, and hexadecapole moment, as shown in Table 2.
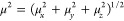
7
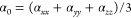
8
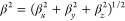
9where
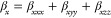
10
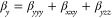
11
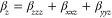
12and
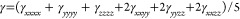
13

**Table 2:** Calculated Linear and NLO Properties of the **TrSR1** Molecule Using B3PW91/LANL2DZ. (Acetone ε = 20.7, Methanol ε = 32.6, and Isopropanol ε = 19.9)

molecule **TrSR1**	*Μ* e.s.u (×10^–18^)	α e.s.u (×10^–26^)	β e.s.u (×10^–34^)	γ e.s.u (×10^–42^)
sample in isopropanol	2.86	–305.55	97.45	–5576.93
sample in acetone	2.87	–305.57	97.57	–5599.75
sample in ethanol	2.89	–305.60	97.90	–5600.41

The dipole characteristics of **TrSR1** are almost similar in all three solvents and are revealed by a nonzero value (Table 2). The values of the dipole moment (μ) are 2.86, 2.87, and 2.89 D in isopropanol, acetone, and ethanol, respectively. A similar trend is also observed for quadrupole moment, octupole moment, and hexadecapole moment values in all solvents, which follow the previously discussed trend.^55^ We performed the same calculations for the other two compounds, which all had the same values in all three solvents. These results suggest that these derivatives are promising NLO candidates for different solvents.

Remarkably, the experimentally obtained 2PA coefficient (β) values of **TrSR1–3** molecules are comparable or better than recently reported graphene-based materials,^48,56^ ferrites,^47^ liquid crystals,^36^ semiconductor nanoparticles,^57^ and alloys.^58^ The optical limiting threshold measured in **TrSR3** (4.5 J/cm^2^) is comparable to recently reported optical limiting threshold values in organic materials and closer to the values of benchmarked materials like carbon-black (2.2 J/cm^2^), C_60_ (2 J/cm^2^),^59^ and considerably lower than Pt (33.1 J/cm^2^) and Pd (24.2 J/cm^2^) nanoparticles.^60^ A literature comparison of the 2PA coefficient (β) values and optical limiting values is summarized in Table 3.

**Table 3:** Literature Comparison of Nonlinear Optical Properties with **TrSR3**

material	two-photon absorption coefficient (β) (m/W)	optical limiting threshold (J/cm^2^)	reference
**TrSR3**	7.9 × 10^–10^	4.5	**this work**
truxene DLC		4.8	(36)
polyfluorene		5	(61)
oligofluorene T6		3.1	(22)
rGO	0.8 × 10^–14^	4.7	(48)
Ag/β-MnO_2_	6.9 × 10^–10^	1.67	(62)
CdSe/CdS/ZnS QDs	0.1 × 10^–10^		(63)
(E)-*N*′-(4-chlorobenzylidene)-4-hydroxybenzohydrazide	0.65 × 10^–11^		(64)
benzene derivative	1.2 × 10^–12^		(65)
l-methionine barium bromide (LMBB)	1.7 × 10^–11^		(66)
4-methylalanilinium 3.5-dinitrobenzoate (MADNBA)	5.8 × 10^–10^		(67)

## 4 Conclusions

This work provides a simple systematic strategy to synthesize truxene-based derivatives for efficient optical limiting applications. We have demonstrated that 2PA and 3PA induced optical limiting in three different truxene derivatives under 5 ns and 100 fs excitations, respectively. The dipole moment, quadrupole moment, octupole moment, and hexadecapole moment were calculated by using DFT calculations. The compound **TrSR3** exhibited a lower optical limiting threshold of 4.5 J/cm^2^ owing to the elongated π-conjugation and branching effect, which is comparable to the optical limiting efficiency of benchmark materials like carbon black and C_60_, liquid crystals, and organic semiconductors. The analysis demonstrated that enlarging the π-branch size and including thiol-derivatives units in the truxene core effectively enhance the NLO properties.

## Supporting Information Available

^1^H NMR spectra of all the derivatives (PDF)
