# Supplementary material for: Nonlinear Optical Properties of Discotic Hexylthiotruxene Derivatives
Source: ACS Omega. 2023 Nov 18;8(48):45961–9. doi: 10.1021/acsomega.3c06778 (PMC10702317; doi:10.1021/acsomega.3c06778)
Supplement: Supplementary file 1 — ao3c06778_si_001.pdf [file ao3c06778_si_001.pdf]

Supporting information

Nonlinear Optical Properties of Discotic Hexylthiotruxene Derivatives

Manish Kumar[#*1], Sreekanth Perumbilavil[#§2], DR Vinayakumara[#*2], Alok Goel[3,4], Reji Philip[2], and Sandeep Kumar[*2,5]

[1]University of Turku, Department of Mechanical and Materials Engineering, FI-20014 Turku, Finland.

[2]Raman Research Institute, C.V. Raman Avenue, Bangalore - 560 080, India.

[3]Institute of Polymer Nanotechnology (INKA), FHNW University of Applied Sciences and Arts Northwestern Switzerland, School of Engineering, Klosterzelgstrasse 2, 5210 Windisch, Switzerland.

[4]Laboratory for Surface Science and Technology, Department of Materials, ETH Zurich, Switzerland.

[5]Department of Chemistry, Nitte Meenakshi Institute of Technology, Yelahanka, Bangalore – 560064, India

[#]Authors contributed equally

*** Corresponding author**

[§] Present address: Centre for Optical and Laser Engineering, School of Mechanical and Aerospace Engineering, Nanyang Technological University, 50 Nanyang Avenue, 639798, Singapore.

1. **NMR Characterization**

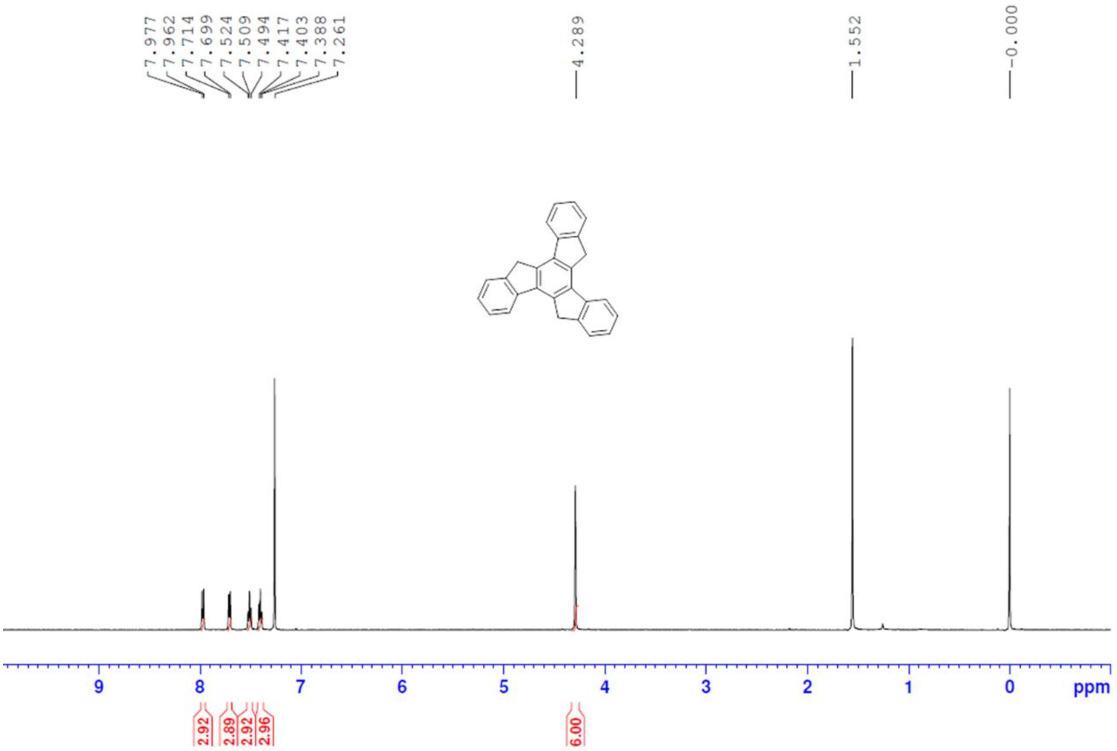

Figure S1: ^{1}H NMR (500 MHz) spectrum of **2** recorded in $CDCl_3$

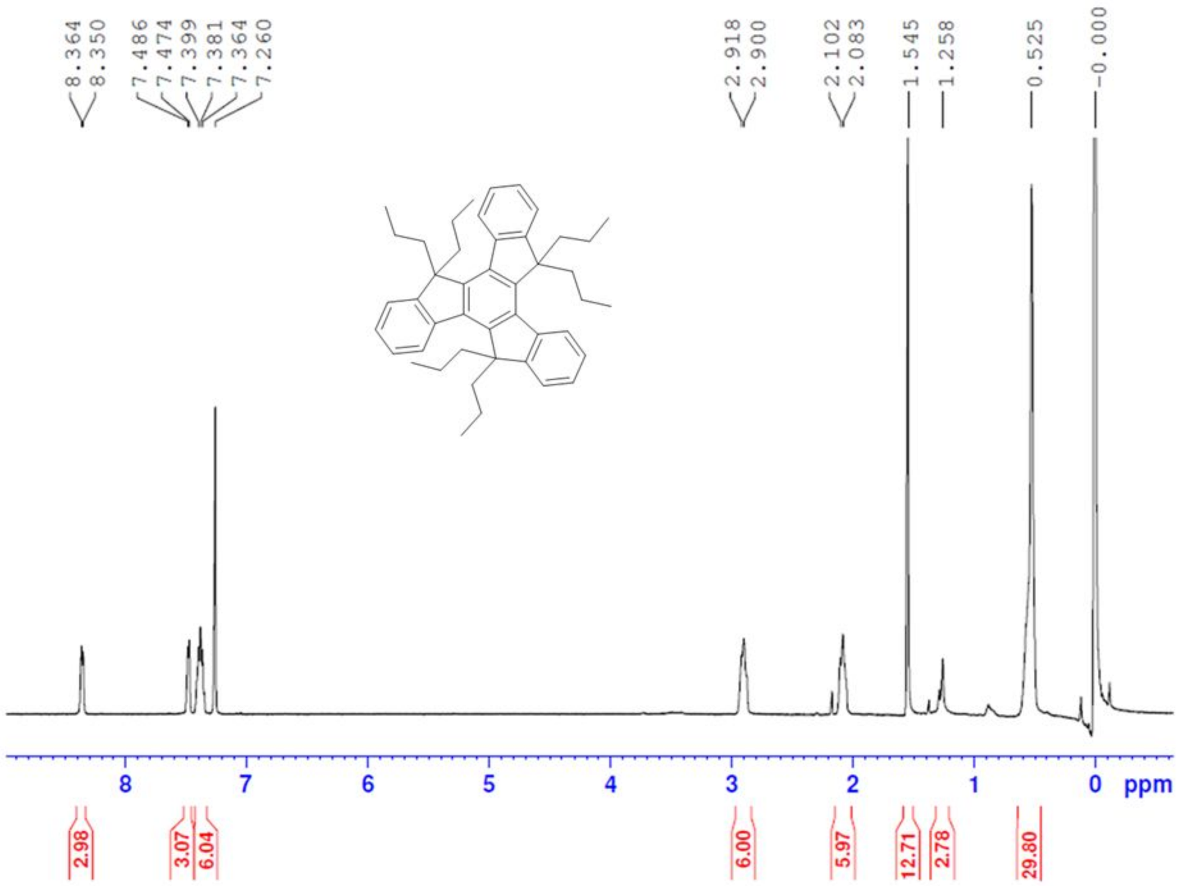

Figure S2: ^{1}H NMR (500 MHz) spectrum of **3** recorded in $CDCl_3$

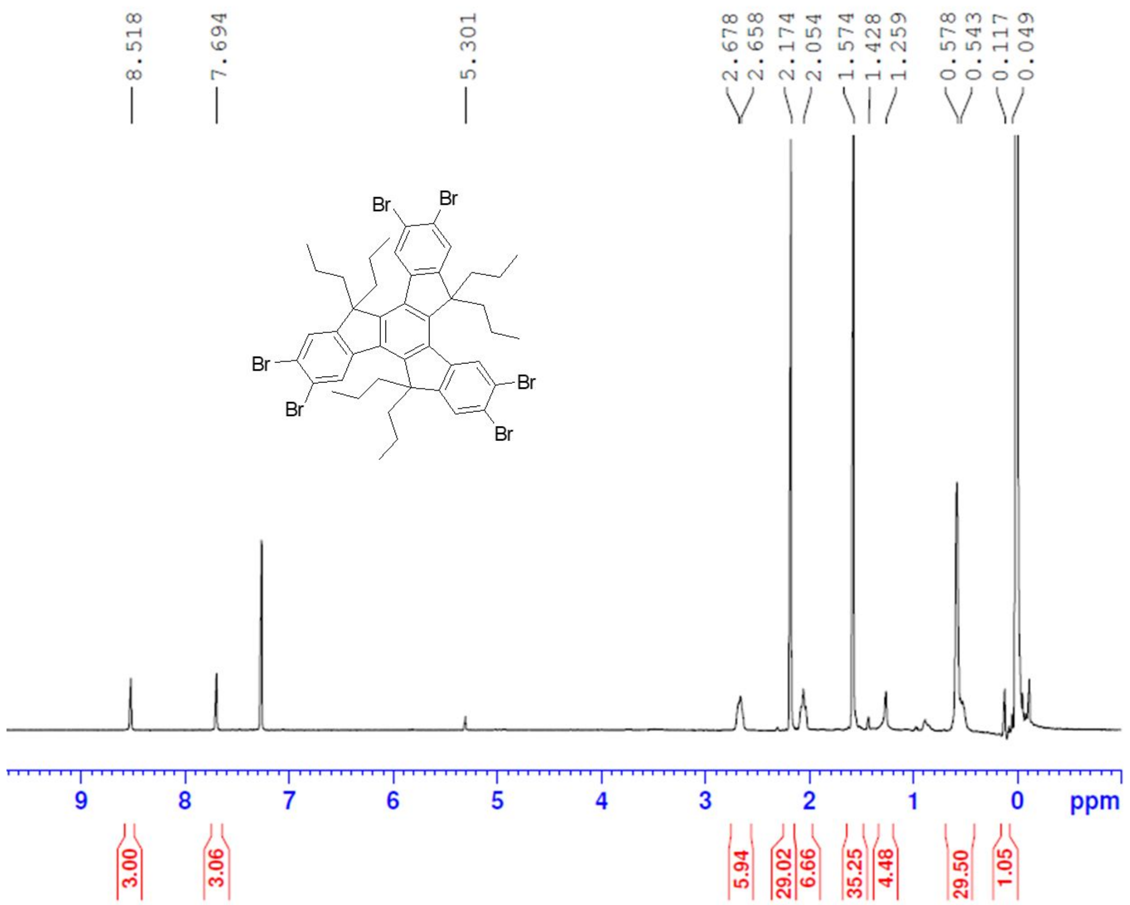

Figure S3: ^{1}H NMR (500 MHz) spectrum of **4** recorded in $CDCl_3$

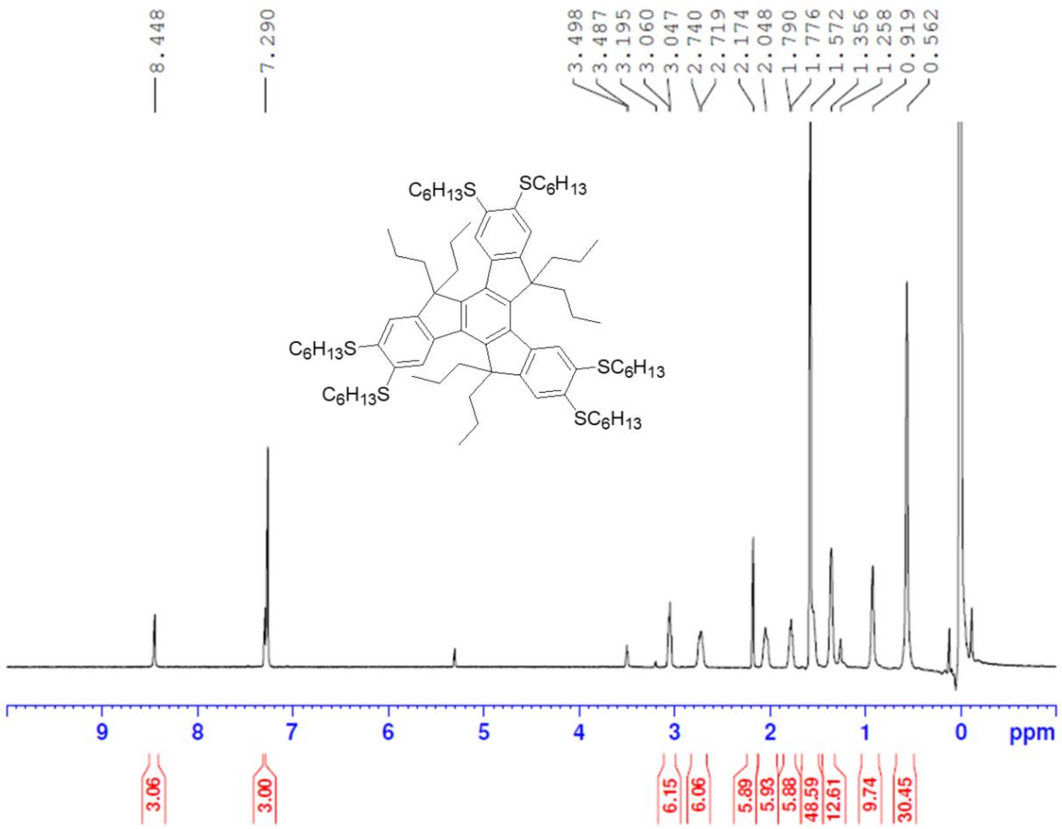

Figure S4: ^{1}H NMR (500 MHz) spectrum of **TrSR1** recorded in $CDCl_3$

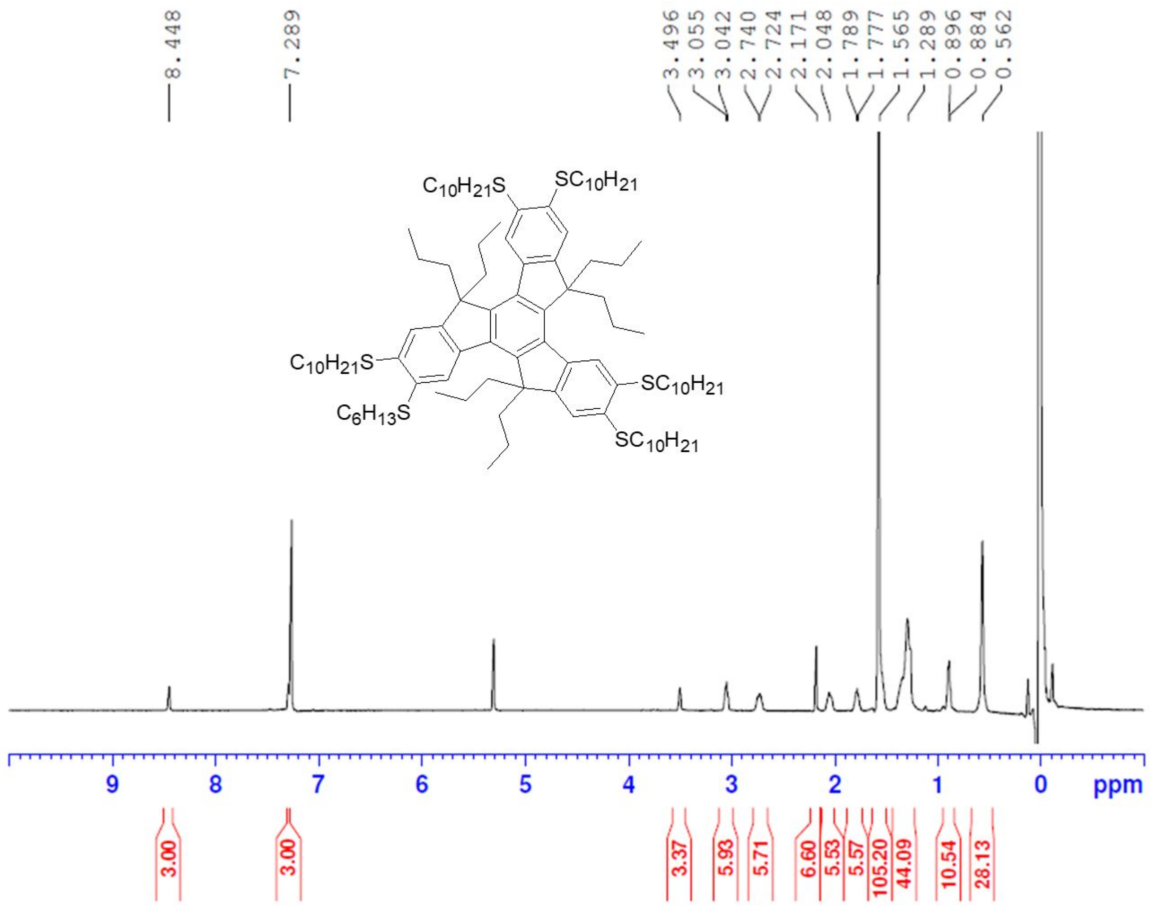

Figure S5: ^{1}H NMR (500 MHz) spectrum of **TrSR2** recorded in $CDCl_3$

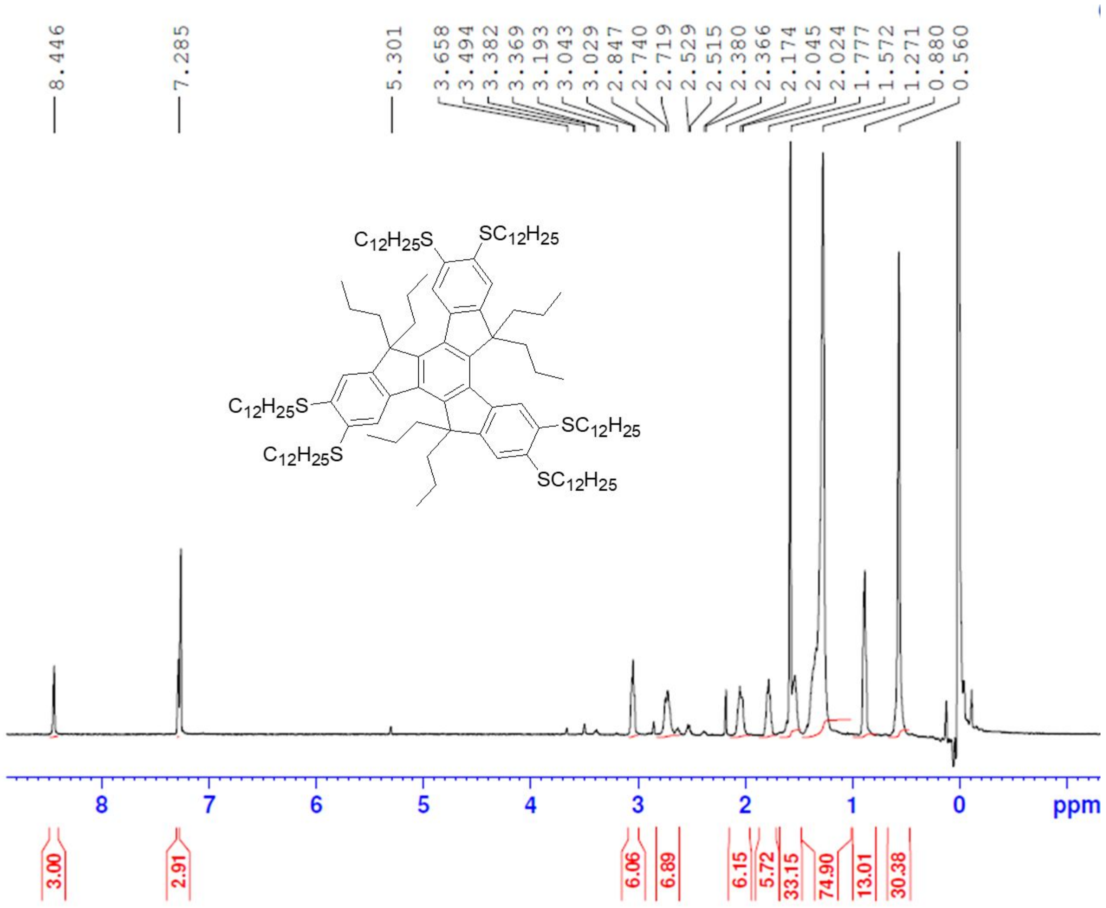

Figure S6: ^{1}H NMR (500 MHz) spectrum of **TrSR3** recorded in $CDCl_3$
